# First person – Mitchell Booth

**DOI:** 10.1242/bio.059586

**Published:** 2022-09-09

## Abstract

First Person is a series of interviews with the first authors of a selection of papers published in Biology Open, helping researchers promote themselves alongside their papers. Mitchell Booth is first author on ‘ Tissue-specific transcriptome profiles identify functional differences key to understanding whole-plant response to life in variable salinity’, published in BiO. Mitchell is a PhD student in the lab of Elizabeth Sinclair at the University of Western Australia Oceans Institute, using omics to understand the mechanisms of life in a changing world.


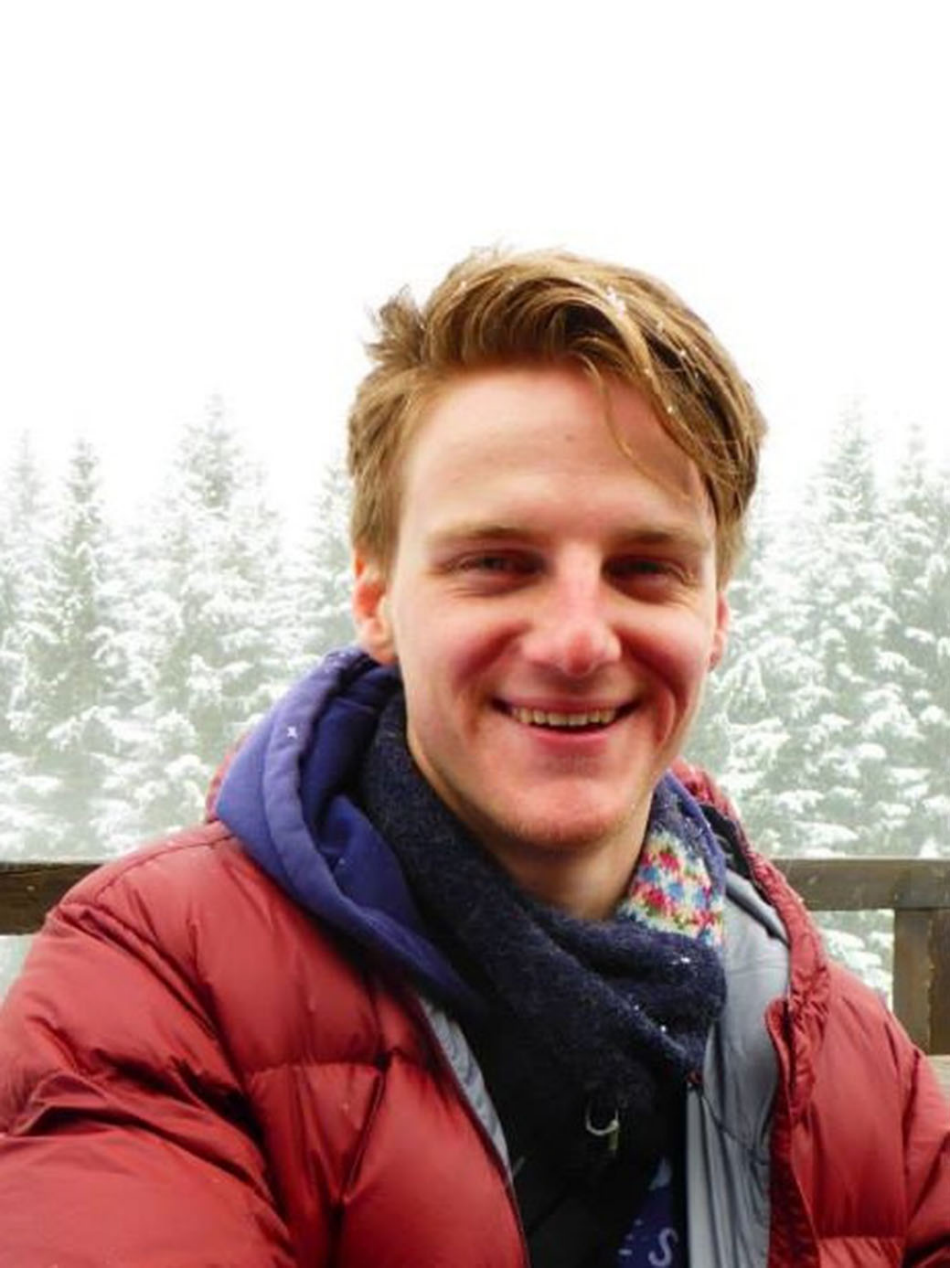


**Mitchell Booth**

**Describe your scientific journey and your current research focus**

I have been interested in biology since high school, however it wasn't until university where I found a real passion toward the application of genetics and omics to understand biological systems for the betterment of society and the environment. My formal research has involved a broad taxon of biological systems and scope of genetics/omics research. As an Honours student I characterised transcriptomic changes in the paralysis tick, *Ixodes holocyclus*, for the advancement of control methods. I then changed systems to human cancers; working initially as a research assistant, assessing novel chemotherapies and CRISPR applications in childhood leukaemia; and later as a data manager, curating information for brain, lung and breast cancer clinical trials. Now I am completing a PhD, investigating transcriptomic changes in the seagrass, *Posidonia australis*, to inform conservation and restoration management of the World Heritage Site, Shark Bay, in Western Australia. My experience has exposed me to many wet-lab and bioinformatic analyses, which has shaped my overarching research focus of using genetics and omics to unravel the complexity of at-risk biological systems.

**Who or what inspired you to become a scientist?**

I consider myself very lucky to have had a passionate biology teacher at the public high school I attended. He really inspired me to question and dig deeper to understand the reason why things are the way they are.

**Figure:**
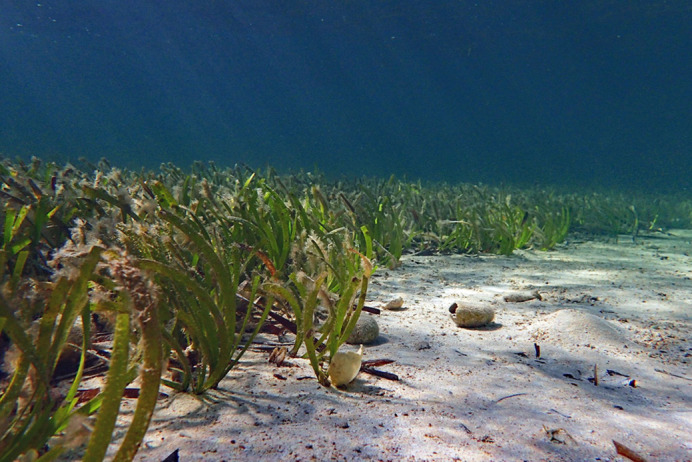
The long-lived seagrass, *Posidonia australis*, a major marine ecosystem engineer in the Shark Bay World Heritage Site, WA. Photo by Rachel Austin, UWA.

**How would you explain the main finding of your paper?**

By analysing leaf, basal leaf meristem and root tissues of the seagrass, *Posidonia australis*, we were able to show that both the group of genes being activated, and the extent of activation, differed significantly between tissues and between geographical sites across Shark Bay. The tissue differences were explained by the different primary roles each tissue plays the plant: leaves are the main site of photosynthesis, basal leaf meristems focus on leaf tissue growth, and roots focus on sediment nutrient absorption. The differences between geographical sites, however, were instead explained by the salinity gradient occurring in both gulfs of Shark Bay; where *P. australis* growing in southern, more saline waters showed more signs of salinity regulation processes than those growing in the northern, less saline waters.

**What are the potential implications of this finding for your field of research?**

These results show the *in situ* gene expression patterns of a temperate seagrass, naturally growing at its range edge in a highly biodiverse location. The biological processes identified provide insight into seagrass life in variable salinity, as well as highlighting the importance of considering basal leaf meristem when evaluating whole-plant responses to environmental change.
